# Gut microbiota dysbiosis and decreased levels of acetic and propionic acid participate in glucocorticoid-induced glycolipid metabolism disorder

**DOI:** 10.1128/mbio.02943-23

**Published:** 2024-01-16

**Authors:** Qin Zhang, Gaopeng Guan, Jie Liu, Wenmu Hu, Ping Jin

**Affiliations:** 1 Department of Endocrinology, The Third Xiangya Hospital, Central South University, Changsha, Hunan, China; Cornell University, Ithaca, New York, USA

**Keywords:** gut microbiota, glucocorticoids, SCFAs, glycolipid metabolism

## Abstract

Long-term/high-dose glucocorticoid (GC) use results in glycolipid metabolism disorder, which severely limits its clinical application. The role of the gut microbiota and its metabolites in GC-induced glycolipid metabolism disorder remains unclear. Our previous human study found that obvious gut microbiota dysbiosis characterized by an increasing abundance of *Proteobacteria* and a decreased abundance of *Lachnospiraceae* and *Faecalibacterium* were observed in patients with endogenous hypercortisolism. In this study, we established a mouse model of GC-induced glycolipid metabolism disorder (Dex group) and found that the relative abundances of *Proteobacteria* and *Parasuttrerella* were increased, while the abundances of *Lachnospiraceae*, *Faecalibacterium*, and *Lachnospiraceae_NK4A136_group* were decreased significantly in the Dex group. Compared with the control group, serum total short-chain fatty acids (SCFAs), acetic acid, propionic acid, and GLP-1 levels were all decreased in the Dex group. The mRNA expression of the GPR41 receptor and Pcsk1 in the colon was significantly decreased in the Dex group. Furthermore, GC-induced glycolipid metabolism disorder could be alleviated by depletion of the gut microbiota or fecal bacteria transplantation with control bacteria. The abundances of *Lachnospiraceae_NK4A136_group* and the serum GLP-1 levels were significantly increased, while the abundances of *Proteobacteria* and *Parasutterella* were significantly decreased after fecal bacteria transplantation with control bacteria. Our work indicates that gut microbiota dysbiosis and decreased levels of serum acetic acid and propionic acid may participate in GC-induced glycolipid metabolism disorder. These findings may provide novel insights into the prevention and treatment of GC-induced metabolic disorders.

**IMPORTANCE:**

The role of the gut microbiota in glucocorticoid (GC)-induced glycolipid metabolism disorder remains unclear. In our study, gut microbiota dysbiosis characterized by an increased abundance of *Proteobacteria*/*Parasuttrerella* and a decreased abundance of Lachnospiraceae_NK4A136_group was observed in mice with GC-induced glycolipid metabolism disorder. Some bacteria were shared in our previous study in patients with endogenous hypercortisolism and the mouse model used in the study. Furthermore, the depletion of the gut microbiota and fecal bacteria transplantation with control bacteria could alleviate GC-induced glycolipid metabolism disorder. Plasma acetic acid, propionic acid, and GLP-1 and the mRNA expression of the GPR41 receptor and Pcsk1 in the colon were decreased significantly in mice with GC-induced glycolipid metabolism disorder, which indicated that the gut microbiota/SCFA/GPR41/GLP-1 axis may participate in GC-induced glycolipid metabolism disorder. Our findings indicate that the gut microbiota may serve as a novel therapeutic target for GC-related metabolic disorders.

## INTRODUCTION

Glucocorticoids (GCs) have been widely used in a number of clinical states because of their anti-inflammatory and immunosuppressive effects. However, long-term or high-dose application of GCs can lead to glycolipid metabolism disorders, including abdominal obesity, hyperglycemia, hyperlipidemia, and more (1, 2), which severely limits their clinical application. The mechanism of GC-induced glycolipid metabolism disorder is still unclear. As reported, GCs can reduce the secretion of glucagon-like peptide-1 (GLP-1), which can promote glucose-induced insulin secretion, increase satiety, and reduce obesity (3). GLP-1 is a gastrointestinal hormone produced in intestinal epithelial endocrine L-cells by the differential processing of proglucagon. Studies have shown that the gut microbiota can regulate the production of GLP-1 through the short-chain fatty acid (SCFA)/G protein-coupled receptor (GPCR) signaling pathway (4, 5).

The gut microbiota plays a crucial role in maintaining the physiological functions of the host by regulating body metabolism and immunity (6, 7). Imbalances in the gut microbiota and its metabolites are closely related to obesity, metabolic syndrome, and diabetes (8). To date, reports on the gut microbiota and GC-induced glycolipid metabolism disorders are rare. Xie et al. (9) found that the abundance of *Bacteroidetes* was significantly decreased, whereas the abundance of *Firmicutes* and *Proteobacteria* was increased in mice administered corticosterone for 28 days. Zhang et al. observed that the relative abundance of Lachnospiraceae decreased in rats treated with prednisone (10 mg/kg/day) for 6 weeks (10). However, it is difficult to accurately determine the impact of glucocorticoids on the gut microbiota in patients treated with glucocorticoids. The duration and the dosage of glucocorticoid usage vary greatly based on the disease, and undoubtedly those variables may interfere with the characteristics of the gut microbiota. To date, there was only one human study that analyzed the gut microbiota in 28 patients with acute transverse myelitis who received prednisone (50–60 mg/day) for 7 days and found a dramatic decrease in gut microbial diversity, with enrichment of Firmicutes and depletion of *Bacteroidetes* in GC-induced obese individuals (11). In contrast, patients with Cushing’s syndrome have a prolonged increase in endogenous glucocorticoids, the condition of which offers an opportunity to better reflect the influence of glucocorticoids on gut microbiota disorders. Thus, we investigated the gut microbiota and plasma short-chain fatty acids (SCFAs) in patients with endogenous hypercortisolism (Cushing’s syndrome; 12). We found that the α diversity of the gut microbiota was dramatically decreased in patients with endogenous hypercortisolism. Compared to controls, the patients with endogenous hypercortisolism were enriched in *Proteobacteria* and depleted in Lachnospiraceae and *Faecalibacterium* (12).

SCFAs are an important metabolite of the gut microbiota and can improve glucose and lipid metabolism, control energy expenditure, and regulate the immune system and inflammatory responses (13). However, reports on SCFAs and GC-induced glycolipid metabolism disorders are rare. Our previous study found that the level of propionic acid was significantly decreased in patients with endogenous glucocorticoid excess (12). Qiu et al. also found that SCFA content was decreased in GC-induced obese individuals (11). Zhang et al. (10) observed that the level of propanoic acid decreased in rats treated with prednisone. Thus, we intended to clarify the causal relationship and the possible mechanism underlying the gut microbiota and GC-induced glycolipid metabolism disorder in mouse models.

## RESULTS

### Mouse model of GC-induced glycolipid metabolic disorder

**Fig 1:**
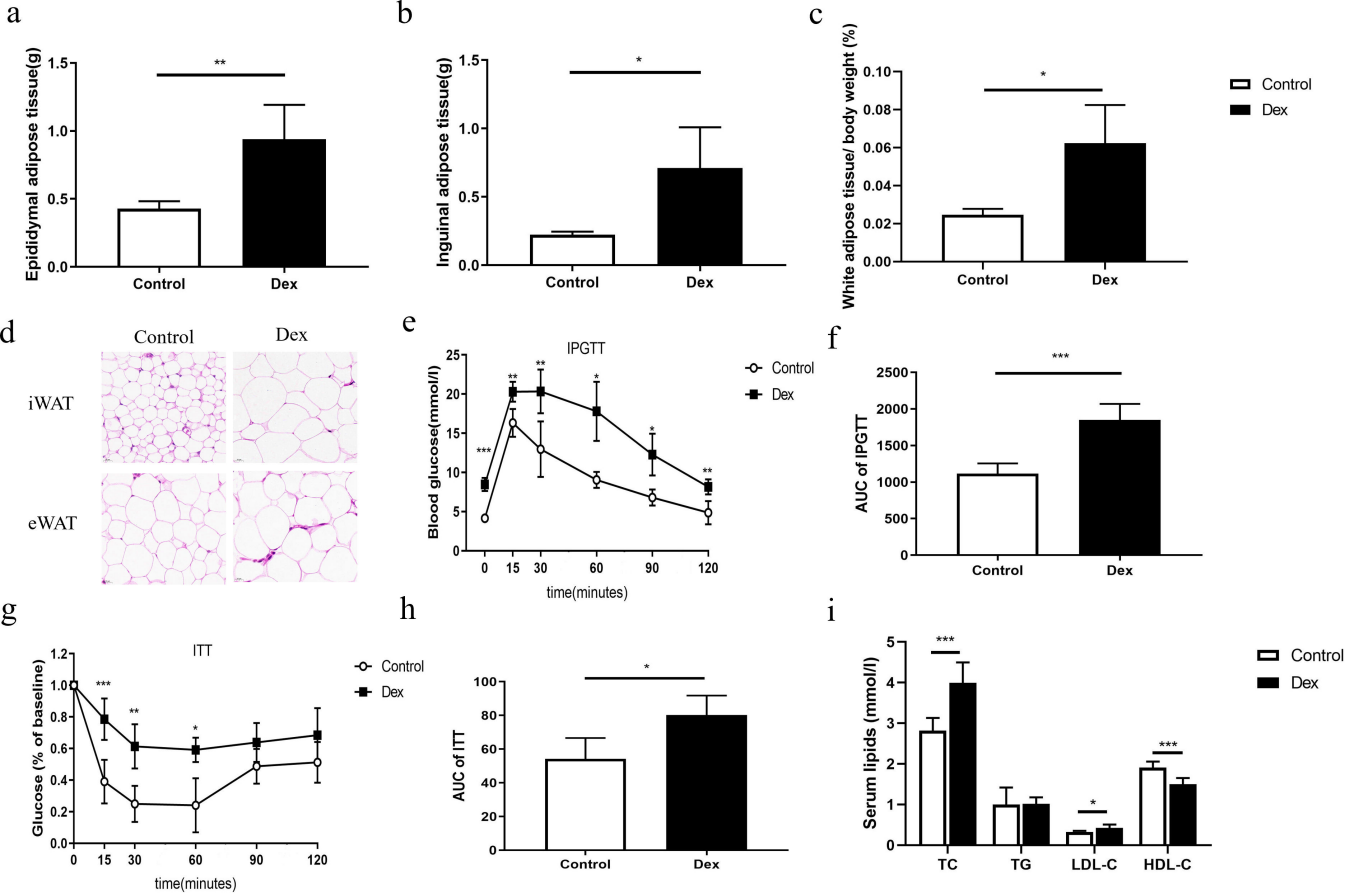
Mouse model of GC-induced glycolipid metabolism disorder. (a–c) Compared to controls, the contents of inguinal and epididymis white adipose tissue and the ratio of white adipose tissue to net body weight were increased in the Dex group. (d) HE staining showed that the lipid droplet of adipose tissue of mice was significantly increased in the Dex group (the magnification of each field is ×40, and the lower left ruler is 20 µm). (e and f): The Dex group showed worse glucose intolerance in the IPGTT test. (g and h) T The Dex group mice showed higher insulin resistance compared to those of control mice. (i) Compared to controls, Dex group mice showed lipid disorders. iWAT, inguinal white adipose tissue; eWAT, epididymal white adipose tissue. Dex group: mice with glucocorticoid-induced metabolic disorder (*n* = 6); control group (*n* = 6). IPGTT, intraperitoneal glucose tolerance test; ITT, insulin tolerance test; AUC, area under the curve. Compared to controls, *P < 0.05, **P < 0.01, and ***P < 0.001.

Compared to controls, the contents of white adipose tissue, such as inguinal and epididymal adipose tissue, and the ratio of white adipose tissue to net body weight of mice were significantly higher in the Dex group (Fig. 1a through c, *P* < 0.05). The adipocyte volumes of inguinal white adipose tissue and epididymal white adipose tissue of mice were also significantly increased in the Dex group (Fig. 1d). Compared to controls, the blood glucose level and area under the IPGTT curve of mice in the Dex group were significantly higher (Fig. 1e and f, *P* < 0.01), while the insulin sensitivity in the ITT test was significantly decreased in the Dex group (Fig. 1g and Fig. 1h, *P* < 0.05). Compared to controls, Dex mice had higher levels of TC and LDL-C and lower levels of HDL-C (Fig. 1i, *P* < 0.05). All the above results indicated that the mouse model of GC-induced glycolipid metabolic disorder was established successfully.

### Characteristics of the gut microbiota in mice with GC-induced glycolipid metabolic disorder

**Fig 2:**
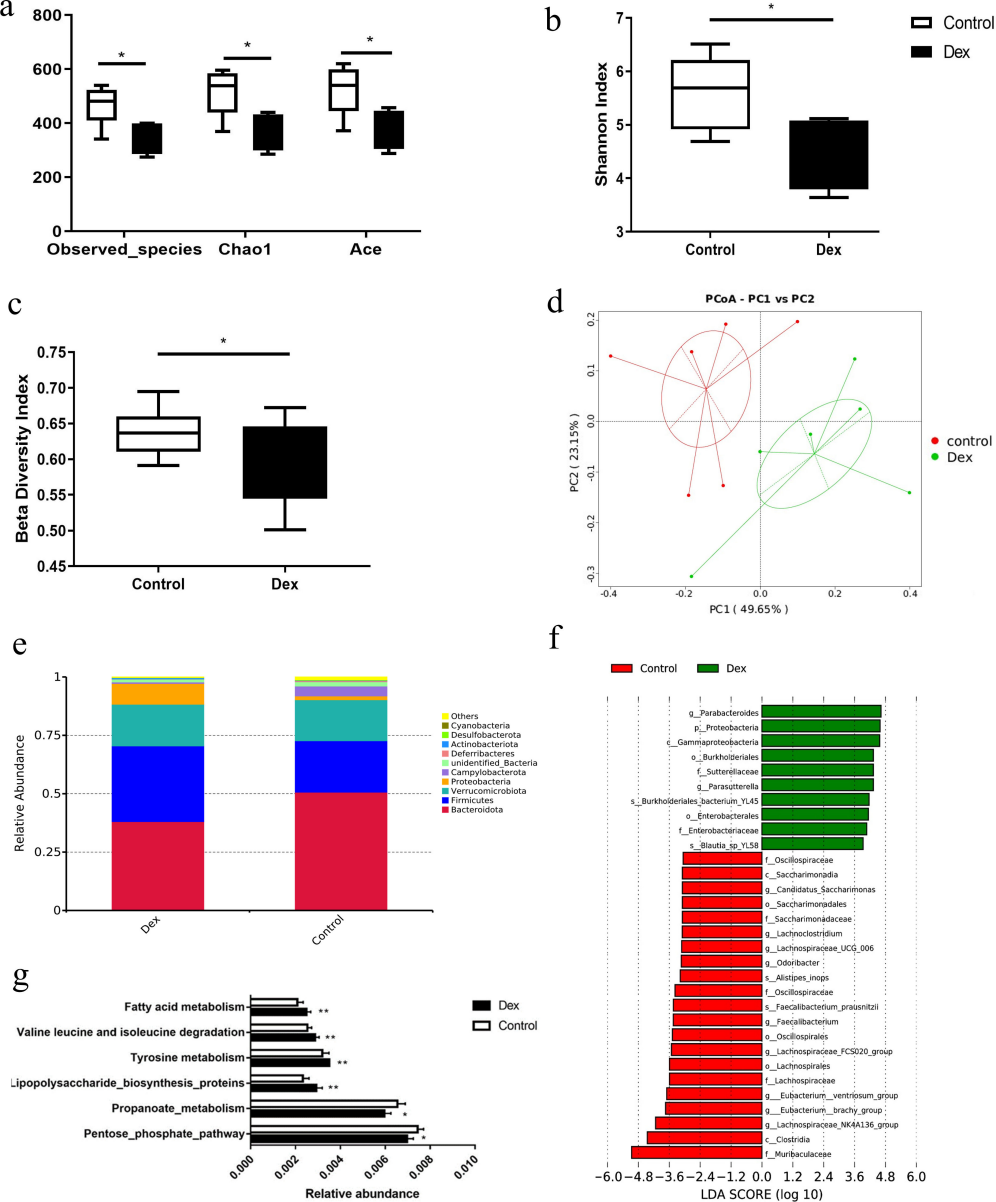
Characteristics of the gut microbiota in mice with GC-induced glycolipid metabolism disorder. (a and b) The α diversity indices were significantly decreased in the Dex group compared to controls. (c) The β diversity significantly decreased in the Dex group compared to controls. (d) PCoA analysis showed that the gut microbiota of the Dex group clustered separately from the control group. (e) The relative abundance of gut bacteria at the phylum level between the Dex and control group. (f) LEfSe analysis of gut microbiota between the Dex and control group. (g) PICRUST analysis of gut microbiota between the Dex and control groups. Compared to controls, *P < 0.05. *n* = 6.

Compared with controls, the α diversity indices, including the Observed_species index, Chao1 index, Shannon index, and ACE index, were significantly decreased in the Dex group, which indicated that the diversity and evenness of gut microbiota in GC-induced metabolic disorder mice were significantly decreased (Fig. 2a and b). The β diversity of the gut microbiota in Dex mice was also significantly decreased (Fig. 2c). The bacterial community composition of the Dex group was distinct from that of control mice according to principal coordinate analysis (PCoA) based on weighted UniFrac distances (Fig. 2d). There was a significant difference in the gut microbiota community structure of Dex and control mice by Adonis analysis (*R*^2^ = 0.382, *P* = 0.01), ANOSIM analysis (*R* = 0.604, *P* = 0.018), and MRPP analysis (*A* = 0.1274, *P* = 0.007; Table. S1). Compared with the controls, the relative abundance of *Proteobacteria* in the Dex group was significantly increased (8.9% vs 1.6%, *P* = 0.009; Fig. 2e). LEfSe analysis (LDA >3) further showed that the relative abundances of *Gammaproteobacteria*, *Enterobacteriaceae*, *Sutterellaceae*, *Parasuttrerella*, and *Parabacteroides* increased significantly, while the relative abundances of *Lachnospiraceae*, *Lachnospiraceae_NK4A136_group*, *Lachnospiraceae_UCG_006*, *Eubacterium_ventriosum _group*, *Eubacterium_brachy_group*, *Faecalibacterium*, *Faecalibacterium_prausnitzii*, and *Odoribacter* decreased significantly in the Dex group (Fig. 2f, *P* < 0.05). PICRUST analysis revealed that the abundance of lipopolysaccharide synthetic protein pathway genes was significantly increased, while propanoate metabolism and pentose phosphate pathway genes related to propionic acid synthesis were significantly decreased in the Dex group (Fig. 2g, *P* < 0.05). To analyze the correlation between different bacteria and fasting blood glucose and white fat content, Spearman correlation analysis was conducted at the phylum level and genus level. *Proteobacteria* and *Parasutterella* were positively correlated, while *Lachnospiraceae_NK4A136_group* and *Odoribacter* were significantly negatively correlated with iWAT and eWAT and fasting blood glucose levels (Fig. S1a and S1b, *P*< 0.05).

### Changes in SCFAs and correlation with gut bacteria

**Fig 3:**
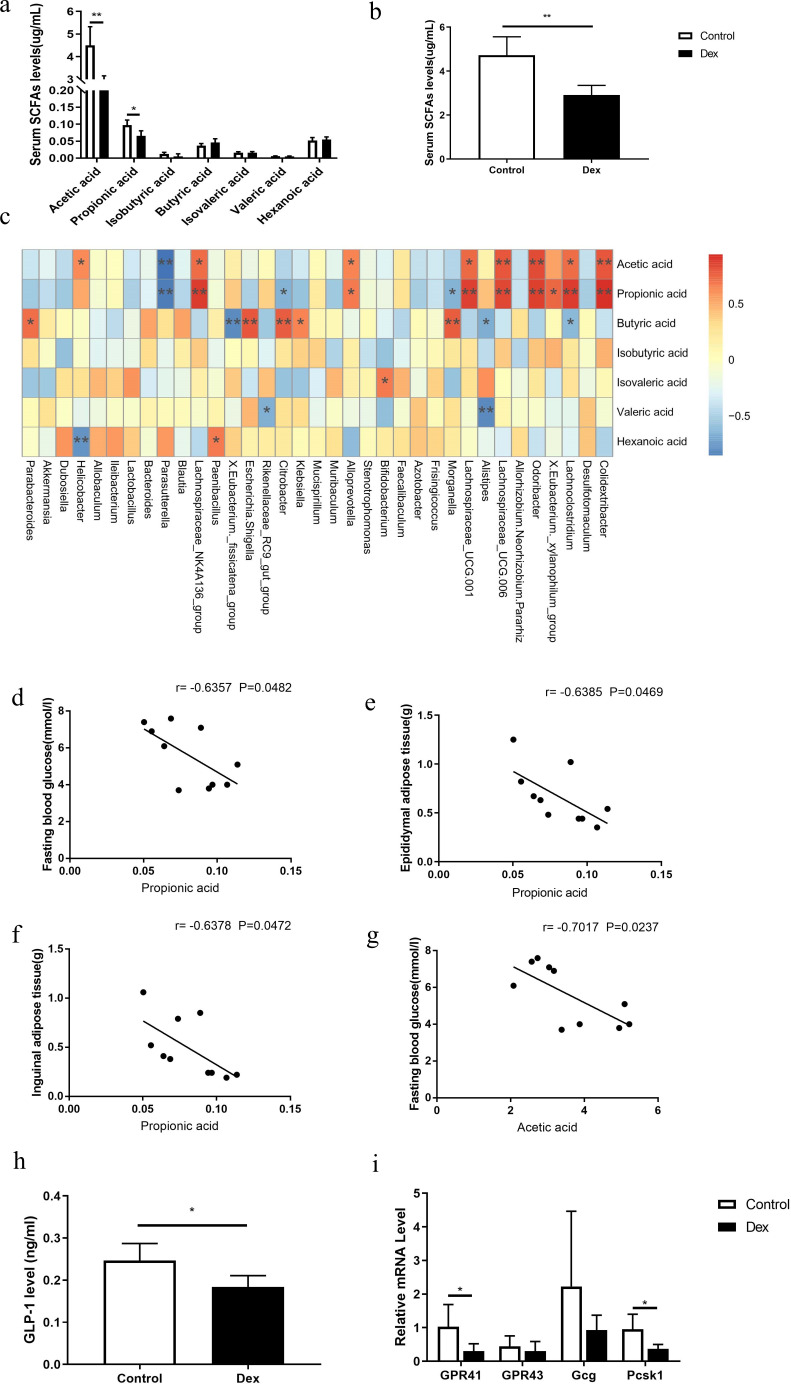
Changes in SCFA and GLP-1 levels in mice with GC-induced glycolipid metabolism disorder. (a and b) The serum total SCFA, acetic acid, and propionic acid levels were significantly decreased in the Dex group. (c) Spearman correlation analysis of SCFAs and different gut bacteria. (d–g) Spearman correlation analysis of propionic acid and acetic acid with fasting blood glucose, eWAT, and iWAT. (h) The serum GLP-1 level in Dex and control mice. (i) The mRNA levels of GPR41 and Pcsk1 in the colon of the Dex and control groups. Compared to controls, *P < 0.05; **P < 0.01. *n* = 6.

In the above results, we observed a decrease in the abundance of short-chain fatty acid-producing bacteria, including *Lachnospiraceae*, *Lachnospiraceae_NK4A136_group*, *Lachnospiraceae_UCG_006*, *Eubacterium* sp., *Faecalibacterium*, and *Faecalibacterium prausnitzii*, in the Dex groups. Thus, we further detected the serum levels of short-chain fatty acids in the Dex and control groups by GC-MS. Compared with the controls, the levels of acetic acid (4.51 ± 0.82 vs 2.72 ± 0.43 µg/ml, *P* = 0.005), propionic acid (0.10 ± 0.02 vs 0.07 ± 0.01 µg/ml, *P* = 0.011), and total serum short-chain fatty acids (4.73 ± 0.84 vs 2.91 ± 0.44 µg/ml, *P* = 0.005) decreased significantly in the Dex group (Fig. 3a and b). Spearman correlation analysis showed that the contents of acetic acid and propionic acid were positively correlated with *Lachnospiraceae_NK4A136_group*, *Odoribacter*, and *Lachnospiraceae_UCG-006* (Fig. 3c, *P* < 0.05). The propionic acid levels were significantly negatively correlated with eWAT and iWAT contents and fasting blood glucose, while acetic acid was significantly negatively correlated with fasting blood glucose levels (Fig. 3d and g, *P* < 0.05).

### GLP-1 levels in mice with GC-induced glycolipid metabolic disorder

The serum level of GLP-1 in Dex group mice was significantly lower than that in control mice (0.25 ± 0.04 vs 0.18 ± 0.03 ng/ml, *P* = 0.021, Fig. 3h). Studies have shown that both acetic acid and propionic acid reduce GLP-1 secretion through colon GPCRs (14–16). Thus, we detected the mRNA level of colon GPCRs and found that the mRNA expression level of GPR 41 was significantly decreased in the Dex group compared to the control group (Fig. 3i, *P* < 0.05). The mRNA expression level of the proprotein convertase subtilisin/kexin type 1 (Pcsk1) gene, which acts as an enzyme to cut and process proglucagon to produce GLP-1, was also significantly decreased in the Dex group compared to the control group (Fig. 3i, *P* < 0.05).

### Depletion of the gut microbiota could alleviate GC-induced glycolipid metabolism disorder

**Fig 4:**
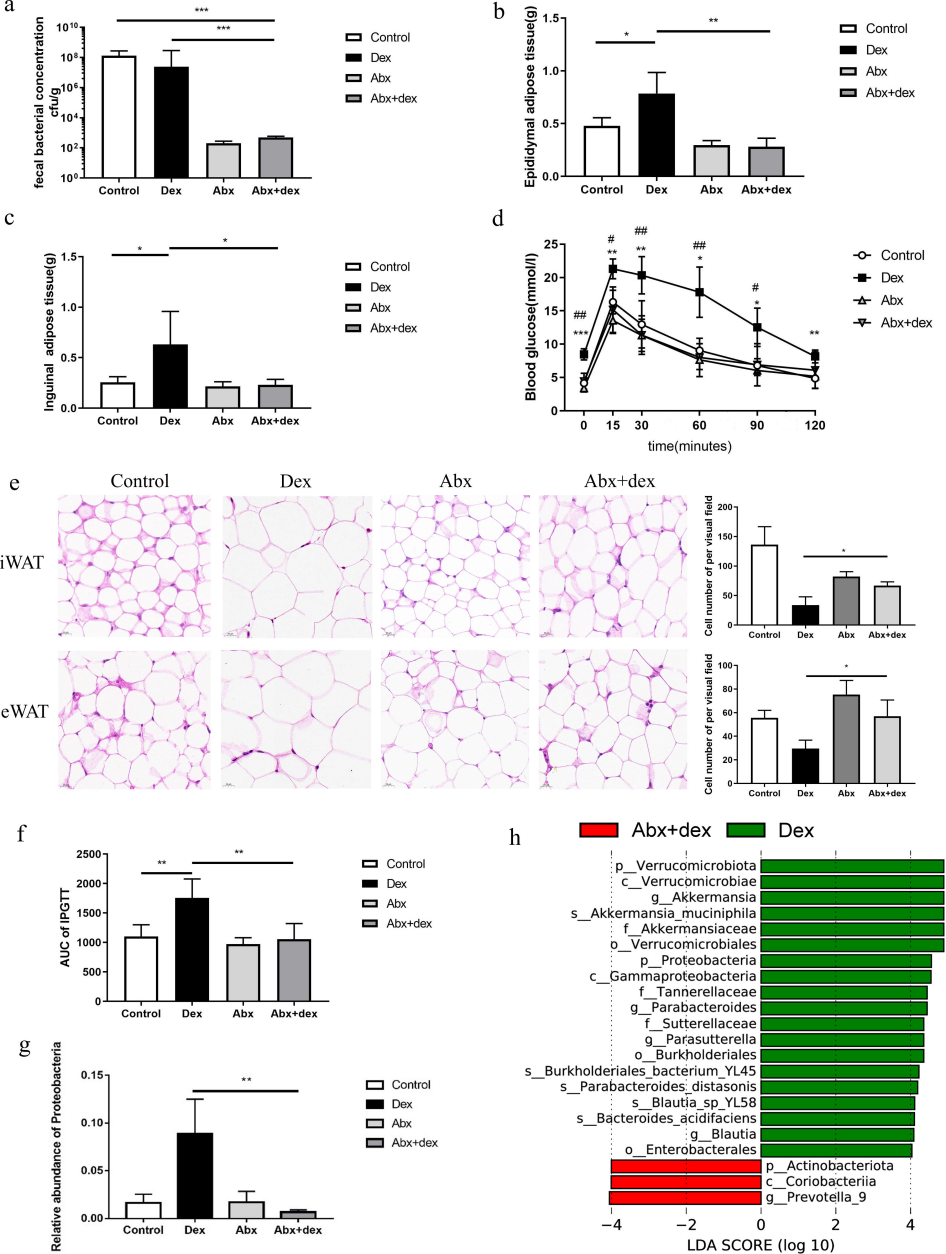
Depletion of the gut microbiota could partly rescue GC-induced glycolipid metabolism disorder. (a) Antibiotic treatment eliminated most of the gut bacteria in the Abx + dex and Abx mice. (b–c) The eWAT and iWAT contents of the Abx + dex group were significantly decreased compared with those of the dex groups. (d–f) The IPGTT showed that the Abx + dex group had better glucose tolerance. (e) HE staining showed that the Abx + dex group had fewer lipid droplets than the Dex group; (g) The relative abundance of *Proteobacteria* in the Abx + dex and Dex groups. (h) LEfSe analysis of the Abx + dex and Dex groups. Dex (*n* = 6); control (*n* = 6); Abx (*n* = 6); Abx +dex (*n* = 6). Compared to Abx +dex, *P < 0.05, **P < 0.01, ***P < 0.001. *n* = 6.

To verify the causal relationship between the gut microbiota and GC-induced glycolipid metabolism disorder, we investigated the effects of GC on glycolipid metabolism after depletion of the gut microbiota. After the 14th day of antibiotic treatment, most of the gut bacteria were eliminated in the Abx + dex and Abx mice compared with the control and dex groups (Fig. 4a). After efficient microbial depletion, the epididymal adipose tissue and inguinal adipose tissue of Abx +dex mice were lower than those of the Dex group (Fig. 4b and c, *P* < 0.05). The IPGTT test showed that the blood glucose and the area under the curve of the Abx +dex group were significantly lower than those of the Dex group (Fig. 4d and f, *P* < 0.05). Compared to the Dex group, HE staining showed that the lipid droplets in white adipose tissue were decreased in the Abx +dex group (Fig. 4e). There were no differences in the amount of white adipose tissue or glucose tolerance among the Abx, Abx + dex, and control groups. 16S rRNA sequence analysis showed that the relative abundance of *Proteobacteria* in Abx + dex mice was lower than that in the Dex group (Fig. 4g). LEfSe analysis showed that *Proteobacteria*, *Parasutterella*, and *Parabacteroides* were decreased in the Abx + dex mice compared with those in the Dex group (Fig. 4h).

### GC-induced glycolipid metabolism disorder improved by fecal bacteria transplantation

**Fig 5:**
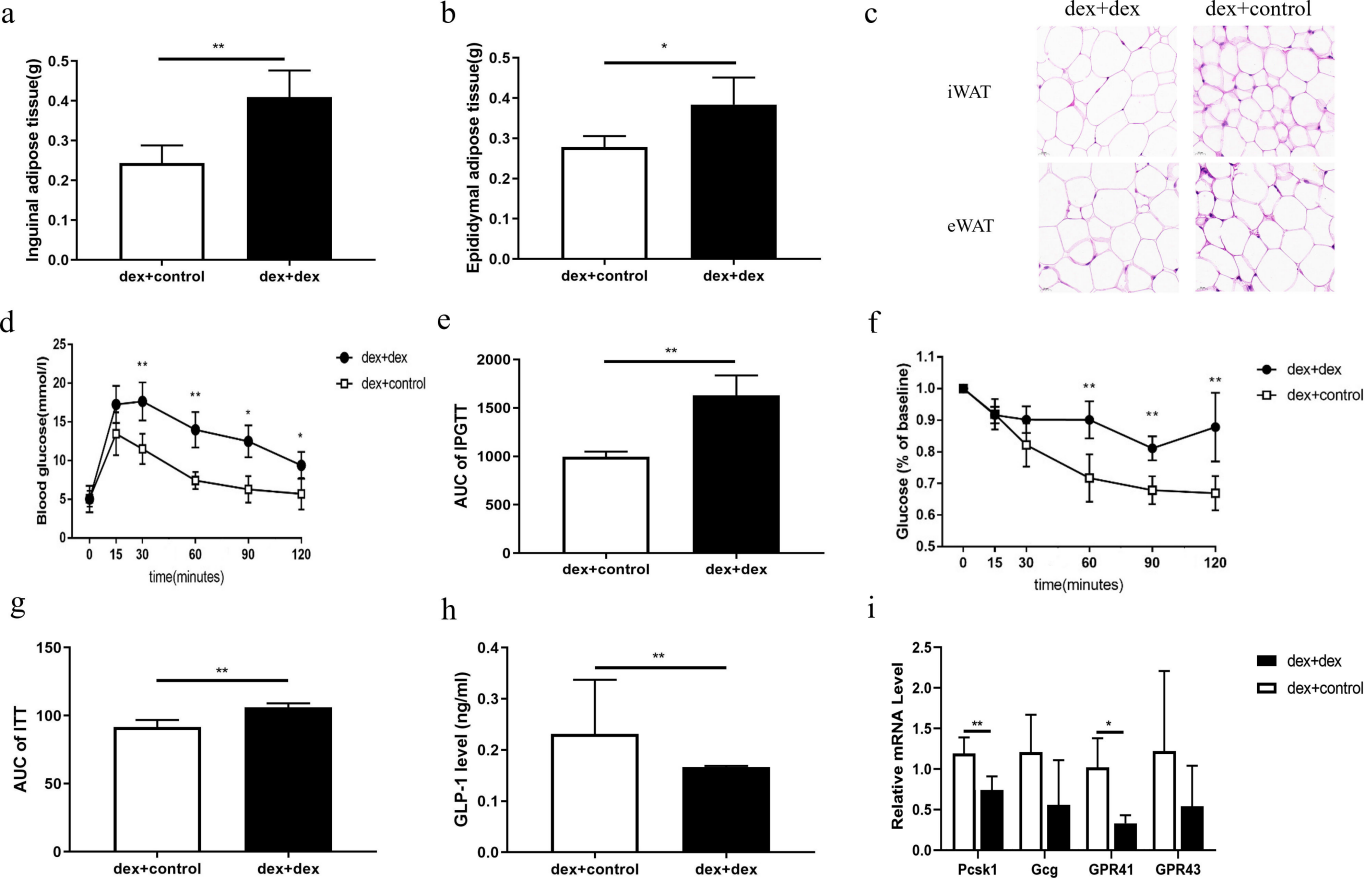
Fecal bacteria transplantation in mice with GC-induced metabolic disorder. (a and b) Compared to the dex + dex group, the dex + control group had lower eWAT and iWAT contents. (c) HE staining of white adipose tissue. (d and e) IPGTT test showed that the dex + control group had better glucose tolerance. (f and g) ITT test showed that the dex + control group had better insulin sensitivity. (h) Serum GLP-1 in the dex + control group was higher than that of the dex + dex group. (i) The mRNA expression levels of the GPR41 and Pcsk1 genes of dex + control mice were significantly higher than those of dex + dex mice. Compared to the dex + dex group, *P < 0.05, **P < 0.01, ***P < 0.001. *n* = 6.

Feces from control mice and Dex group mice were intragastrically administered to C57/BL6J mice to observe whether fecal bacteria transplantation of control mice feces could alleviate GC-induced glycolipid metabolism disorder. Compared with the dex + dex group, the epididymal adipose tissue and inguinal adipose tissue of the dex + control mice were significantly decreased (Fig. 5a and b, *P* < 0.05). HE staining showed that the lipid droplets of white fat in the dex + control group were also significantly decreased compared with those in the controls (Fig. 5c). Compared with the dex + dex group, the blood glucose and the AUC area under the IPGTT curve in the dex + control group were significantly decreased (Fig. 5d and e, *P* < 0.05). Insulin resistance in the ITT test improved significantly in the dex + control group (Fig. 5f and g, *P* < 0.05). In addition, the serum GLP-1 level (0.23 ± 0.11 vs 0.17 ± 0.01 ng/ml, *P* = 0.006) and the mRNA expression levels of the colon GPR41 and Pcsk1 genes were increased in the dex + control group compared with those in the dex +dex group (Fig. 5h and i, *P* < 0.05).

### Characteristics of gut microbiota after fecal bacteria transplantation

**Fig 6:**
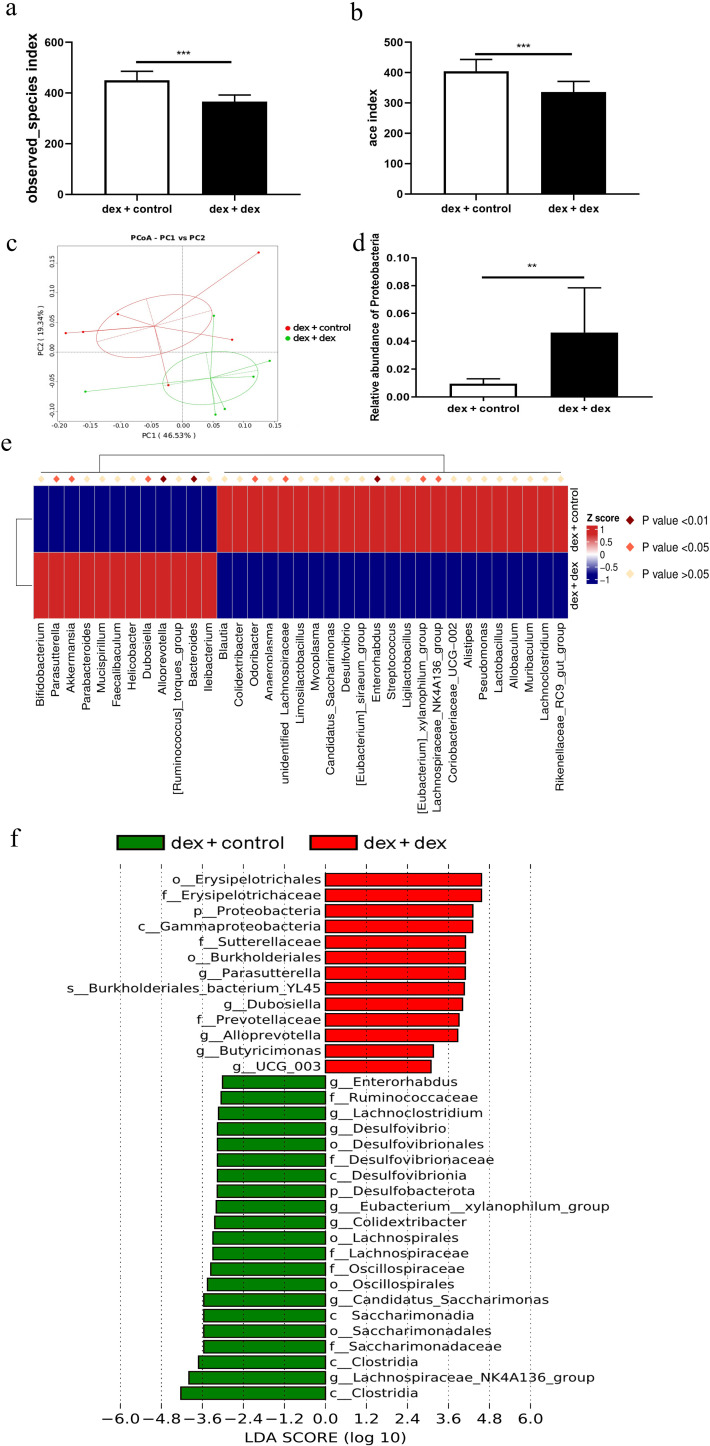
Characteristics of the gut microbiota after fecal bacteria transplantation. (a and b) The α diversity index of the dex + control group increased significantly. (c) PCoA of the dex + dex group and the dex + control group. (d) The relative abundance of *Proteobacteria* in the dex + control mice was significantly reduced compared with that in the dex + dex mice. (e and f) MetaStat analysis and LEfSe analysis of the dex + dex and dex + control mice. Compared to dex + dex mice, **P < 0.01, ***P < 0.001. *n* = 6.

To identify the gut microbiota characteristics of recipient mice after fecal bacteria transplantation and the key bacterial genera that regulate glycolipid metabolism, 16S rRNA sequencing was performed in the feces of mice after fecal bacteria transplantation. Compared to those of the dex + dex mice, the observed species index and ACE index of the dex + control mice were significantly higher, indicating that transplantation with control feces could restore the decline in gut microbiota diversity induced by GCs (Fig. 6a and b, *P* < 0.05). PCoA showed that the samples of the dex + dex group were distributed far away from those of the dex + control group, indicating that there were significant differences in gut microbiota structure between these two groups (Fig. 6c, *P* < 0.05). Compared to that in the dex + dex mice, the relative abundance of *Proteobacteria* in the dex + control group mice was significantly reduced (Fig. 6d, *P* < 0.05). MetaStat and LEfSe analysis showed that the relative abundance of *Parasutterella* was significantly decreased, while the relative abundance of *Lachnospiraceae_NK4A136_group* was significantly increased in the dex + control group (Fig. 6e and f, *P* < 0.05).

## DISCUSSION

Long-term/high-dose glucocorticoid use results in glycolipid metabolism disorder, but the role of gut microbiota in GC-induced glycolipid metabolism disorder is still unknown. In our study, we observed noticeable gut microbiota dysbiosis in mouse models with GC-induced glycolipid metabolism disorder. Interestingly, some bacteria were shared in the patients with endogenous hypercortisolism and the mouse model used in this study, such as the increased abundance of *Proteobacteria* and decreased abundance of short-chain fatty acid-producing bacteria, including *Lachnospiraceae*, *Eubacterium* spp., and *Faecalibacterium* (12). The level of serum propionic acid also decreased significantly in both the patients and the mouse model. Furthermore, the depletion of the gut microbiota and fecal bacteria transplantation with control bacteria could alleviate glucocorticoid-induced glycolipid metabolism disorder, which verified that the gut microbiota plays a crucial role in GC-induced glycolipid metabolism disorder.

Consistent with our previous study in patients with endogenous hypercortisolism (12), the relative abundances of *Proteobacteria* were significantly increased in mice with GC-induced glycolipid metabolism disorder. As reported, the increase in intestinal *Proteobacteria* was an important feature of the instability of intestinal microbial community structure (17) and was observed in obese type 2 diabetes patients and high-fat diet-fed C57BL/6J obese mice (14, 18). *Proteobacteria* are a group of Gram-negative bacteria that can produce lipopolysaccharide (LPS), which is known to lead to endotoxemia, obesity, insulin resistance, and other metabolic abnormalities. In our study, the LPS synthesis pathway genes of the gut microbiota were increased significantly in the Dex group, which indicated that *Proteobacteria* may take part in GC-induced glycolipid metabolism disorder through the LPS pathway. *Parasutterella* was also increased in mouse models with GC-induced glycolipid metabolism disorder, which was reported to be associated with obesity and diabetes (15, 16). A cohort study from European and American populations found that *Parasutterella* was positively associated with BMI and type 2 diabetes independent of the reduced microbiome α/β diversity and low-grade inflammation commonly found in obesity (19). Recently, researchers successfully isolated and colonized *Parasutterella* in the gastrointestinal tract of mice and found that the level of fecal 5-hydroxytryptophan (5-HT) increased significantly (20). As reported, 5-HT could promote WAT lipid droplet formation and inhibit WAT beige through 5-hydroxytryptophan receptor 2A (5-HTR2A 21, 22).

Another important characteristic of gut microbiota dysbiosis caused by GC was the decreasing abundance of *Lachnospiraceae_NK4A136_group*. Many studies have shown that *Lachnospiraceae_NK4A136_group* is an SCFA-producing bacterium (23, 24). Shiwei et al. (25) and Huizhen et al. (26) observed a decline in the abundance of *Lachnospiraceae_NK4A136_group* in obese patients and high-fat diet-fed mice. The abundance of *Lachnospiraceae_NK4A136_group* was positively correlated with the level of butyric acid and negatively correlated with the level of blood lipids. In addition, increased *Lachnospiraceae_NK4A136_group* abundance was also reported to be associated with the improvement of the metabolic syndrome phenotype in high-fat diet-fed mice treated with polysaccharide sulfate and spermidine (26–28). In addition, the SCFA-producing bacteria *Eubacterium* spp. and *Faecalibacterium* were also found to be decreased significantly in patients with endogenous hypercortisolism and mouse models. Both *Eubacterium* spp. and *Faecalibacterium* were reported to be associated with obesity and diabetes (29, 30). Qiu et al. also found a decline in *Eubacterium* spp. in patients treated with prednisone (11).

SCFAs are important metabolites of the gut microbiota and play an important role in multiple biological processes. In our study, we found that serum total SCFAs, acetic acid, and propionic acid were significantly decreased in the Dex group. Acetic acid and propionic acid are the most common SCFAs. Acetic acid can inhibit white fat decomposition and induce white fat “browning” (31), while propionic acid may reduce the secretion of proinflammatory cytokines, enhance glucose-stimulated insulin release, and inhibit β-cell apoptosis (32). Many studies have shown that acetic acid and propionic acid can regulate the secretion of GLP-1 through GPR41/GPR43 receptors in the gut (33–37). For example, there was a dose-dependent increase in the expression of proglucagon (a precursor of GLP-1) in intestinal endocrine cells cultured with acetic acid (35). Michael et al. observed that rats fed resistant starch had reduced body fat, which may be related to elevated plasma GLP-1 and cecal acetic acid (38). Chambers et al. found that overweight individuals who received direct administration of propionic acid to the proximal colon had increased GLP-1 levels and reduced weight gain (39). In our study, we found that GC-induced glycolipid metabolism disorder mice had decreased plasma GLP-1 and gene transcription of GPR41 and Pcsk1, while plasma GLP-1 and colon mRNA expression of GPR41 and Pcsk1 increased significantly after transplantation with control mouse feces, which indicated that the gut microbiota/SCFA/GPR41/GLP-1 axis may participate in GC-induced glycolipid metabolism disorder. However, the exact mechanism needs to be further confirmed.

In summary, gut microbiota dysbiosis is characterized by an increase in the abundance of *Proteobacteria*/*Parasuttrerella* and a decrease in *Lachnospiraceae*, *Eubacteriu* spp., *Faecalibacterium*, and *Lachnospiraceae_NK4A136_group* may play an important role in GC-induced glycolipid metabolism disorder. Our work provides new ideas for the prevention and treatment of GC-induced metabolic disorders.

## MATERIALS AND METHODS

### Mice and treatment

Ten-week-old male specific pathogen-free (SPF) C57BL/6J mice were purchased from Slake Jingda Experimental Animal Company (China) and housed in an independently ventilated cage at 22 ± 1°C, 55% ± 5% humidity and a light cycle of 12 h a day in the Department Laboratory Animals of Central South University. All the mice were fed with a normal diet (caloric ratio, 12% fat, 66% carbohydrate, 22% protein, 3.5 kcal/g) and sterile water to allow the mice to adapt to the new feeding environment. After 1 week of adaptive feeding, the 11-week-old male C57BL/6J mice were divided into the glucocorticoid induction group (Dex, *n* = 6), in which the mice were injected with 5 mg/kg dexamethasone (Sigma) dissolved in DMSO and then diluted in PBS to a final DMSO concentration of 1%, and the control group (control, *n* = 6), in which the mice were injected with an equal volume of PBS (with 1% DMSO) at 9 a.m. every day for a total of 6 weeks. To determine the effects of glucocorticoid treatment on glucose metabolism, the intraperitoneal glucose tolerance test (IPGTT) and insulin tolerance test (ITT) were conducted as previously reported (40). Blood lipids were detected by an automatic biochemical analyzer. Adipose tissue was fixed with adipose tissue fixating solution including Bouin’s fixating solution and ethanol (Bioss, C2064) for 24 h and dewaxed by embedding sections after dehydration. Then, hematoxylin staining, eosin staining, and dehydration sealing were performed.

### Microbiota depletion experiment

To verify the causal relationship between the gut microbiota and GC-induced glycolipid metabolism disorder, we investigated the effects of GC on glycolipid metabolism after depletion of the gut microbiota by cocktails of antibiotics (Abx). After 1 week of adaptive feeding, 9-week-old male SPF C57BL/6J mice were divided into the following groups. (i) Control group (control, *n* = 6): mice were given ordinary drinking water for 14 days and then injected with the same volume of PBS (with 1% DMSO) for 6 weeks. (ii) Glucocorticoid-induced mice (dex, *n* = 6): mice were given ordinary drinking water for 14 days and then began to be injected with 5-mg/kg dexamethasone (Sigma) for 6 weeks. (iii) Antibiotic-cleaning mice (Abx, *n* = 6): mice were given drinking water mixed with broad-spectrum antibiotics (1 g/L of ampicillin, 1 g/L of neomycin, 1 g/L of metronidazole, and 0.5 g/L of vancomycin, solebaol) for 14 days and then began to be injected with an equal volume of PBS (with 1% DMSO) for 6 weeks. (iv) Glucocorticoid-induced mice treated with antibiotics (Abx + dex, *n* = 6): mice were fed drinking water mixed with broad-spectrum antibiotics for 14 days and then injected with 5-mg/kg dexamethasone for 6 weeks. The fecal samples collected from Abx and Abx + dex group mice after 14 days of antibiotic treatment were plated and cultured on BHI agar with 10% sheep blood. Colony-forming units (CFU/g) were measured to confirm efficient microbial depletion as reported (41).

### Fecal transplant experiment

To further verify the causal relationship between the gut microbiota and GC-induced glycolipid metabolism disorder, we investigated whether fecal bacteria transplantation of control mouse feces could alleviate glycolipid metabolism disorder induced by glucocorticoids. Fresh fecal samples (1 g) were collected from control or Dex group mice and homogenized in 10 mL of prereduced sterile PBS, which was used to gavage the recipient mice. To deplete the original microbiota of the recipient mice, 9-week-old male mice were treated with broad-spectrum antibiotics (1 g/L of ampicillin, 1 g/L of neomycin sulfate, 1 g/L of metronidazole, and 0.5 g/L of vancomycin, Solarbio, China) for 2 weeks prior to the first fecal transplant. After the 14th day of antibiotic treatment, fecal samples of the recipient mice were collected and cultured to confirm efficient microbial depletion. Then, the recipient mice were divided into the following groups. (i) Dex + dex group mice (*n* = 6): mice were injected with 5-mg/kg dexamethasone (Sigma) and gavaged with 200 µl of Dex mouse fecal slurry (3 × 10^5^ CFU) on alternate days for 6 weeks. (ii) Dex + control mice (*n* = 6): mice were injected with 5-mg/kg dexamethasone (Sigma) and gavaged with 200 µl of control mouse fecal slurry (3 × 10^5^ CFU) on alternate days for 6 weeks.

### 16S rRNA sequence analysis

The concrete experimental procedure of the 16S rRNA sequence was conducted as we previously reported (12). PICRUSt was performed to predict microbial metabolic function.

### SCFAs and glucagon-like peptide 1 (GLP-1) analysis

Targeted metabolome analysis of the plasma collected from the different groups of participants was performed using GC-MS (Agilent 7890A/5975C gas-mass spectrometer, Agilent, USA). The specific experimental procedures were described in our previous study (12). The concentration of serum GLP-1 was detected by double-antibody sandwich ELISA (Elabscience, China) and conducted three times.

### RNA extraction and real-time PCR

After grinding, colon RNA was extracted by an RNA extraction kit (TransGen Biotech, China). Reverse transcription and real-time PCR were performed by PerfectStart Green qPCR Super Mix (TransGen Biotech, China) and conducted three times. The primer sequences are shown in Table 1.

**TABLE 1:** Primer sequences

Gene	Primer sequences
β-Actin	Forward: CATCAGCGTAAATGGGGATT; Reverse: AGCTCAGTAACAGTCCGCCTAGA
GPR41	Forward: GACTTGCTCCTGTTGCTGCTG; Reverse: ACTGAACGATGATGACGATGGTG
GPR43	Forward: TACAGTCGCCTGGTGTGGATAC; Reverse: GCCGAAGCAGACGAAGAAGATG
Gcg	Forward: CCTTCAAGACACAGAGG AGAACC; Reverse：CTGTAGTCGCTGGTGAATGTGC
Pcsk1	Forward：GGAGAGAATCCTGTAGGCACCT; Reverse：GCTCTGGTTGAGAAGATGTCCC

### Statistical analysis

SPSS 25.0 statistical software was used for statistical analysis. The normality of distributions was assessed using the Shapiro-Wilk test. Statistical differences in normally distributed continuous variables were evaluated using the Student’s *t* test. Statistical differences in non-normally distributed continuous variables were examined using the Mann-Whitney *U* test. Correlation analysis was performed by Spearman analysis. All tests were bilateral, and *P* < 0.05 was considered statistically significant.

## Data Availability

The original contributions presented in the study are publicly available. The 16S rRNA sequencing presented in the study are deposited in the SRA database, accession number PRJNA1050586. The GC-MS data have been deposited in the MetaboLights database under accession number MTBLS9150. It is anticipated that this accession number will be released by 5 February 2024; until that time, the data will be available from the corresponding author upon request.

## SUPPLEMENTAL MATERIAL

Fig. S1 mbio.02943-23-s0001.tiff Correlation analysis between differentially abundant bacteria and the differential index.

Legend mbio.02943-23-s0002.docx Legend to Fig. S1.

Table S1 mbio.02943-23-s0003.docx Significance test of community structure differences between groups.

ASM does not own the copyrights to Supplemental Material that may be linked to, or accessed through, an article. The authors have granted ASM a non-exclusive, world-wide license to publish the Supplemental Material files. Please contact the corresponding author directly for reuse.
