# Supplementary figures and images for: Gut microbiota dysbiosis and decreased levels of acetic and propionic acid participate in glucocorticoid-induced glycolipid metabolism disorder
Source: mBio. 2024 Jan 16;15(2):e02943-23. doi: 10.1128/mbio.02943-23 (PMC10865841; doi:10.1128/mbio.02943-23)

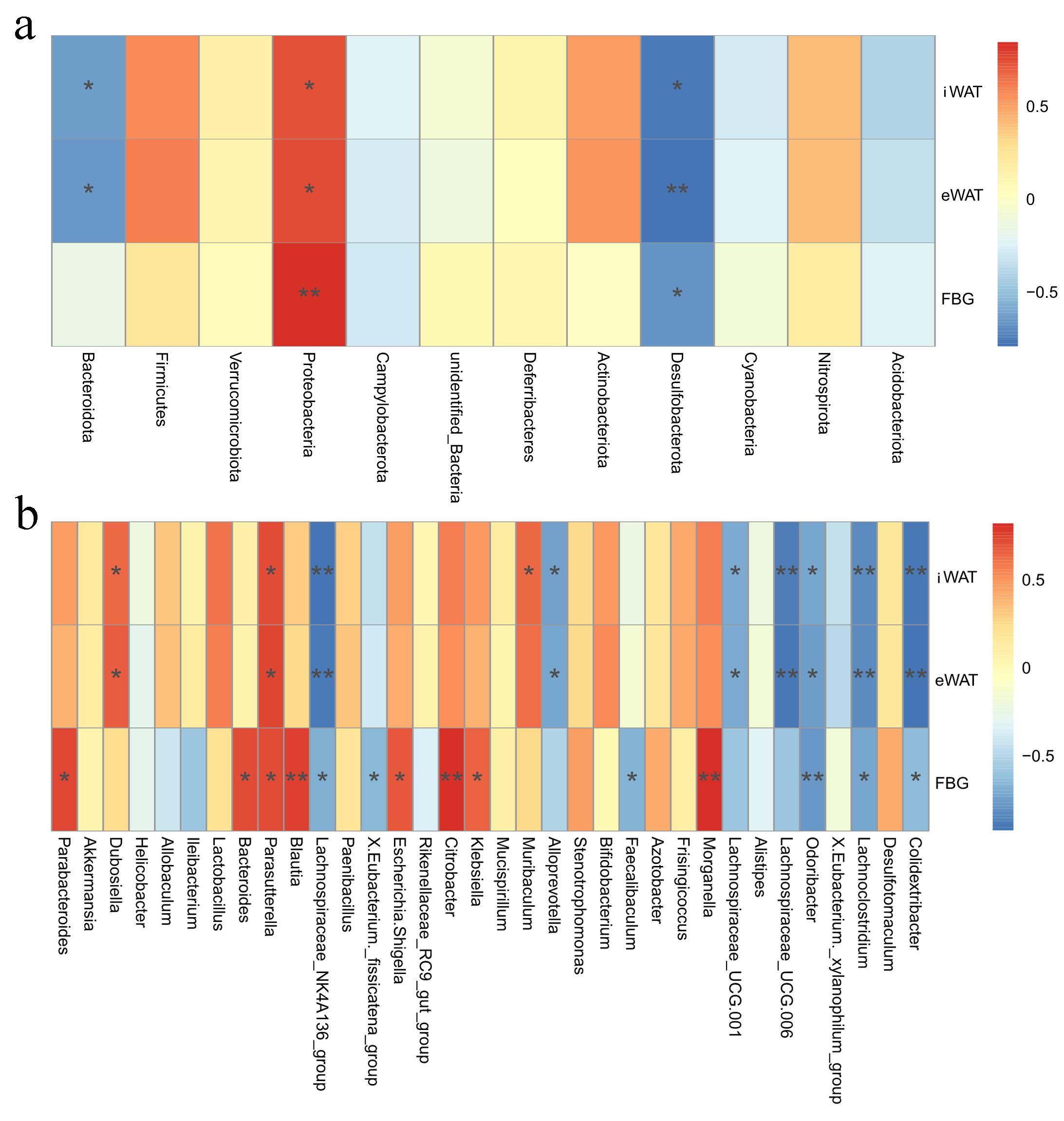

Supplement: Fig. S1 — Correlation analysis between differentially abundant bacteria and the differential index. [file mbio.02943-23-s0001.tiff]
