# Supplementary material for: Gut microbiota dysbiosis and decreased levels of acetic and propionic acid participate in glucocorticoid-induced glycolipid metabolism disorder
Source: mBio. 2024 Jan 16;15(2):e02943-23. doi: 10.1128/mbio.02943-23 (PMC10865841; doi:10.1128/mbio.02943-23)
Supplement: Legend — to Fig. S1. [file mbio.02943-23-s0002.docx]

Figure S1 Correlation analysis between differentially abundant bacteria and the differential index a: Correlation analysis between differentially abundant bacteria at the phylum level and WAT and fasting blood glucose levels; b: Correlation analysis between differentially abundant bacteria at the genus level and WAT and fasting blood glucose levels.
