# Supplementary material for: Gut microbiota dysbiosis and decreased levels of acetic and propionic acid participate in glucocorticoid-induced glycolipid metabolism disorder
Source: mBio. 2024 Jan 16;15(2):e02943-23. doi: 10.1128/mbio.02943-23 (PMC10865841; doi:10.1128/mbio.02943-23)
Supplement: Table S1 — Significance test of community structure differences between groups. [file mbio.02943-23-s0003.docx]

Table S1 Significance test of community structure differences between groups

| Items | R2/R/A/Fs | *P* |
| --- | --- | --- |
| Adonis | 0.38192 | 0.01 |
| Anosim | 0.604 | 0.018 |
| MRPP | 0.1274 | 0.007 |
| Amova | 4.93376 | 0.011 |
